# Intraarticular Administration Effect of Hydrogen Sulfide on an In Vivo Rat Model of Osteoarthritis

**DOI:** 10.3390/ijms21197421

**Published:** 2020-10-08

**Authors:** Carlos Vaamonde-García, Elena F. Burguera, Ángela Vela-Anero, Tamara Hermida-Gómez, Purificación Filgueira-Fernández, Jennifer A. Fernández-Rodríguez, Rosa Meijide-Faílde, Francisco J. Blanco

**Affiliations:** 1 Grupo de Terapia Celular y Medicina Regenerativa, Universidad de A Coruña, Agrupación Estratégica CICA- INIBIC, Complexo Hospitalario Universitario A Coruña, Campus Oza, 15006 A Coruña, Spain; carlos.vaamonde.garcia@udc.es (C.V.-G.); velanero@gmail.com (Á.V.-A.); 2 Grupo de Investigación en Reumatología (GIR), INIBIC-Complexo Hospitalario Universitario A Coruña, Sergas, As Xubias, 15006 A Coruña, Spain; elena.fernandez.burguera@sergas.es (E.F.B.); tamara.hermida.gomez@sergas.es (T.H.-G.); purificacion.filgueira.fernandez@sergas.es (P.F.-F.); 3 Centro de investigación biomédica en Red, Bioingeniería, Biomateriales y Nanomedicina (CIBER-BBN), 28029 Madrid, Spain; 4 Grupo de Envejecimiento e Inflamación, Agrupación Estratégica CICA- INIBIC, Complexo Hospitalario Universitario A Coruña, Sergas, Universidad de A Coruña, As Xubias, 15006 A Coruña, Spain; Jennifer.Fernandez.Rodriguez@sergas.es; 5 Grupo de Investigación de Reumatología y Salud (GIR), Departamento de Fisioterapia, Medicina y Ciencias Biomédicas, Facultad de Fisioterapia, Agrupación Estrategica CICA-INIBIC, Universidade da Coruña, Campus de Oza, 15006 A Coruña, Spain

**Keywords:** hydrogen sulfide, osteoarthritis, oxidative damage, inflammation, Nrf-2

## Abstract

Osteoarthritis (OA) is the most common articular chronic disease. However, its current treatment is limited and mostly symptomatic. Hydrogen sulfide (H_2_S) is an endogenous gas with recognized physiological activities. The purpose here was to evaluate the effects of the intraarticular administration of a slow-releasing H_2_S compound (GYY-4137) on an OA experimental model. OA was induced in Wistar rats by the transection of medial collateral ligament and the removal of the medial meniscus of the left joint. The animals were randomized into three groups: non-treated and intraarticularly injected with saline or GYY-4137. Joint destabilization induced articular thickening (≈5% increment), the loss of joint mobility and flexion (≈12-degree angle), and increased levels of pain (≈1.5 points on a scale of 0 to 3). Animals treated with GYY-4137 presented improved motor function of the joint, as well as lower pain levels (≈75% recovery). We also observed that cartilage deterioration was attenuated in the GYY-4137 group (≈30% compared with the saline group). Likewise, these animals showed a reduced presence of pro-inflammatory mediators (cyclooxygenase-2, inducible nitric oxide synthase, and metalloproteinase-13) and lower oxidative damage in the cartilage. The increment of the nuclear factor-erythroid 2-related factor 2 (Nrf-2) levels and Nrf-2-regulated gene expression (≈30%) in the GYY-4137 group seem to be underlying its chondroprotective effects. Our results suggest the beneficial impact of the intraarticular administration of H_2_S on experimental OA, showing a reduced cartilage destruction and oxidative damage, and supporting the use of slow H_2_S-producing molecules as a complementary treatment in OA.

## 1. Introduction

Osteoarthritis (OA) is the most common articular chronic disease. Despite this high incidence in the population, especially in the elderly, its etiology has not been completely elucidated yet. Likewise, there is no cure for this pathology and its current treatment is limited and mainly focused on alleviating pain, attenuating its progression, and maintaining articular function [1].

Although OA was classically considered to be mainly a consequence of the “wear and tear” of the articular cartilage, we now know that it is a more complex pathology that involves both mechanical and biological processes occurring not only in the cartilage but in the entire joint [1]. In this regard, the increased production of pro-inflammatory mediators in the cartilage and synovial tissue contribute to cartilage destruction that in turn amplifies joint inflammation, creating a vicious circle that contributes to OA development [2,3,4]. Chondrocytes from OA cartilage actively produce pro-inflammatory cytokines, prostaglandins (PGs), or metalloproteinases (MMPs), among others. PGs are key factors in inflammatory processes that are generated by cycloxygenase and PG synthase enzymes after metabolizing arachidonic acid. The expression of inducible isoform of cyclooxygenase, COX-2, is elicited by inflammatory stimuli and oxidative stress [5,6]. This enzyme is responsible for the synthesis of PGE_2_, one of the most important pro-inflammatory prostaglandins in the OA [7]. Additionally, the reactive oxygen species (ROS) production is upregulated in OA, causing oxidative stress. As a result, the byproduct of oxidative damage to proteins, lipids, or DNA could strongly promote the activation of pathological pathways in the joint, including inflammatory and destructive processes [8,9]. Thus, oxidative stress may participate in the pathogenesis of this disease [5]. For instance, the excessive production of nitric oxide (NO) by inducible NO synthase (iNOS) is considered a catabolic event responsible for perpetuating OA pathogenesis by mediating the expression of proinflammatory cytokines, inhibiting the synthesis of the extracellular matrix proteins collagen and proteoglycan [10]. Besides this, NO has been demonstrated to modulate COX-2 expression and prostaglandin production [11] as well as the activation of MMPs in the cartilage [12]. MMPs are well characterized enzymes involved in the degradation of the extracellular matrix (ECM) of the cartilage [13,14]. Among them, MMP-13 (collagenase 3) is an enzyme constitutively expressed in the chondrocyte that degrades collagenous ECM [15]. However, its aberrant expression and activity elicited by pro-inflammatory mediators and oxidative stress has been associated with diseases such as OA [15,16,17].

In relation to OA treatment, disease-modifying drugs have yet to be identified, and the current consensus guidelines recommends the use of a combination of conservative measures, including physical therapy, analgesia, and surgical interventions such as arthroplasty [18]. In the pursuit of an effective and alternative pharmacological treatment for this disease, hydrogen sulfide (H_2_S), an endogenous gas with recognized physiological activities, has emerged as a putative molecule showing a protective effect on different pathological pathways activated in OA cartilage [19,20,21,22]. These actions could be due largely to the role of H_2_S in cellular redox homeostasis. This gas shows antioxidant properties, as it quenches reactive oxygen species (ROS) and reactive nitrogen species (RNS) or increases the expression of antioxidant enzymes by activating the transcription factor nuclear factor erythroid-derived 2-like 2 (Nrf-2), among other mechanisms [23]. Nonetheless, the biosynthesis of this gas is reduced in OA cartilage [24]. Likewise, our group previously detected that the exogenous administration of H_2_S reduced the pro-inflammatory production induced by IL-1β in cultured human chondrocytes, as well as protecting against the degradation of the matrix in ex vivo experiments in OA cartilage explants [19,20]. Additionally, other authors observed in vitro similar actions of the gas in the chondrocyte, such as attenuating inflammatory signaling and exerting chondroprotective effects [21,22,25]. By in vivo experimental approaches, different studies have also detected the beneficial effect of the administration of H_2_S donors on rheumatic disease progression and pain [26,27]. Interestingly, we have recently shown that balneotherapy in sulfur-rich water attenuates the destruction of cartilage and pain levels in an OA model in rats by the surgical destabilization of the joint [28].

Overall, a number of findings suggest the effect of H_2_S-releasing molecules in alleviating symptoms and attenuating progression in different pathologies [29]. However, scarce studies have directly evaluated in vivo the impact of exogeneous H_2_S administration on the signaling pathways involved in the pathogenesis of disorders such as OA. To better understand the potential role of H_2_S as a therapeutic candidate in the treatment of rheumatic diseases, we analyzed in this study the effect of the intraarticular injection of GYY-4137, a slow-releasing H_2_S donor [30], in a surgically induced model of OA in rats.

## 2. Results

### 2.1. Intraarticular Administration of H_2_S Attenuates Joint Pain and Protects against Motor Dysfunction after Experimental OA

**Figure 1:**
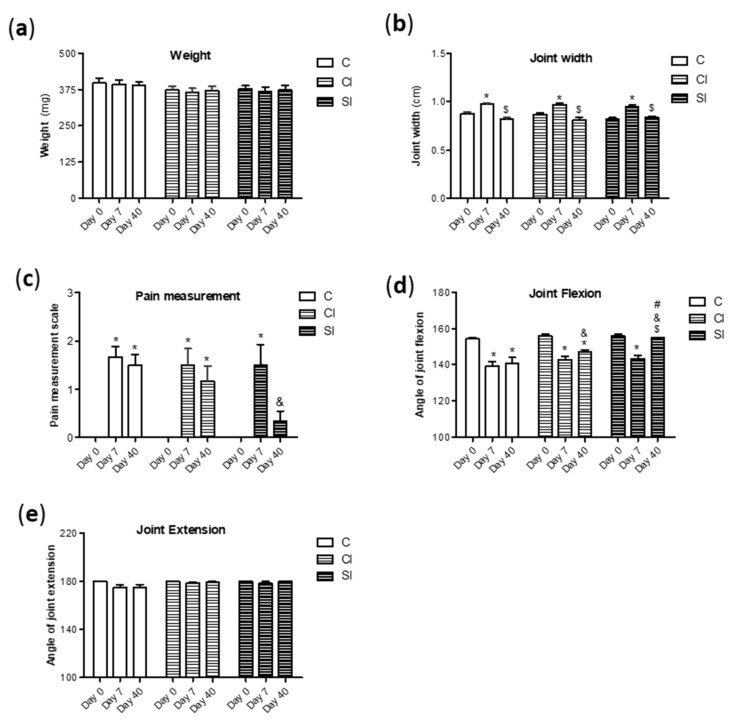
Macroscopic and clinical evaluation. Macroscopic evaluation of the animals from the experimental groups (under non-treatment (C); intraarticular injection of saline (CI) or H_2_S donor (SI)) was performed. Firstly, the weight (**a**) and articular diameter (**b**) were monitored. Evaluation of the pain levels (**c**) was performed with an arbitrary scale, as previously indicated. Finally, the angle of joint flexion (**d**) and extension (**e**) over the course of the model were assessed by a protractor. Values are mean ± SEM (*n* = 6 independent animals for each condition). * *p* ≤ 0.05 vs. day 0; ^$^ *p* ≤ 0.05 vs. day 7; ^&^ *p* ≤ 0.05 vs. the respective day in the control group; ^#^ *p* ≤ 0.05 vs. the respective day in the CI group.

The examination of macroscopic parameters and joint goniometry were completed in order to evaluate the effect of the intraarticular administration of H_2_S on joints with surgically induced lesions. The surgical destabilization of the joint scarcely altered the weight of the rats after the induction of the OA model, observing a recovery over the days (Figure 1a). Additionally, non-differences were observed between groups. As expected, the animals showed joint swelling as an increment of the articular width occurring at day 7 after surgery, which seemed to be recovered at day 40 in the third group (Figure 1b). Besides this, we detected that experimental OA induced at day 7 a significant increment in the level of pain in all experimental conditions (Figure 1c). However, the rats under the H_2_S donor treatment (SI) presented at day 40 significant lower levels of pain than those from the non-treated group (control). This protective effect that was not observed in the group treated with intraarticular saline (CI). Similarly, joint flexion was affected along the tested times in the control and CI groups, but the loss of flexion motion was only transient in the SI group (Figure 1d), observing significant differences at day 40 in comparison with the control and CI group. In contrast, surgical destabilization failed to alter the joint extension at the tested days (Figure 1e).

**Figure 2:**
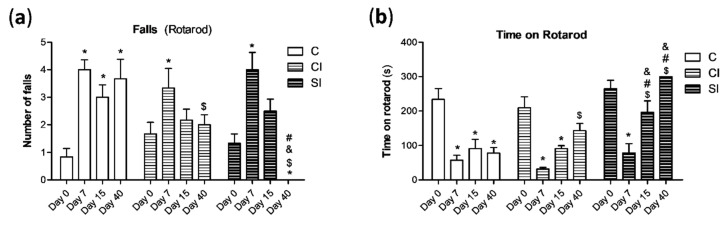
Analysis of the articular motor function with the Rotarod performance test. Animals from the three experimental groups were forced to move in the rotating cylinder for 300 s. The number of falls (**a**) during this period was monitored, as well as the time remaining on the rotarod (**b**). Values are mean ± SEM (*n* = 6 independent animals for each condition). * *p* ≤ 0.05 vs. day 0; ^$^ *p* ≤ 0.05 vs. day 7; ^&^ *p* ≤ 0.05 vs. the respective day in the C group; ^#^ *p* ≤ 0.05 vs. the respective day in the CI group. C, non-treated; CI, control injection; SI, H_2_S donor injection.

We also analyzed the motor function in the animals under experimental OA with the Rotarod performance test. Joint destabilization elicited an initial increment in the number of falls in the Rotarod in all the experimental groups (Figure 2a). Animals from the SI and CI groups showed a significant recovery that was not detected in the control group. Nonetheless, we observed in the SI group that the number of falls at day 40 was significantly lower than that registered at day 7 and that observed at day 40 in the control and CI groups (Figure 2a). Additionally, the time that the rats stayed on the rotating cylinder before the first fall decreased after the OA induction (at day 7) in all the experimental groups (Figure 2b). Once again, the animals treated with the H_2_S donor showed at day 15 and 40 a longer stay on the rotarod than in the control and CI groups at the same days of measurements. Besides this, non-significant differences were detected in the treated group at these mentioned days in comparison with day 0 (Figure 2b), suggesting a clearer and stable recovering under H_2_S treatment.

### 2.2. Intraarticular Administration of H_2_S Protects against Cartilage Destruction Induced by Joint Surgical Destabilization

**Figure 3:**
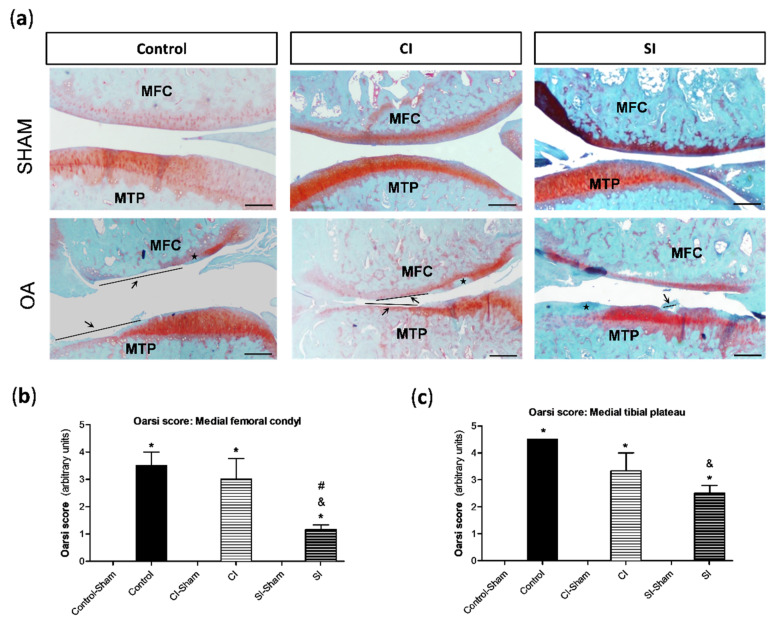
Histological analysis of articular cartilage. Cartilage lesions were evaluated by the semi-quantitative modified osteoarthritis Research Society International (OARSI) score, as previously indicated in the Materials and Methods section. (**a**) Representative images of the joint sections stained with Safranin-O-fast green from each group of the study, showing the cartilage of the medial compartment from the tibial plateau (MTP) and femoral condyle (MFC) in the right knee (sham surgery) and left knee (OA surgery). Arrows indicate areas with a loss of cartilage matrix and ★ indicates cartilage with the loss of Safranin staining (indicator of proteoglycan content). Analysis of the semi-quantitative score of the pathological alterations in the cartilage from MFC (**b**) and MTP (**c**). Values are mean ± SEM (*n* = 3 independent animals for each condition). * *p* ≤ 0.05 vs. the respective sham-operated joint; ^&^ *p* ≤ 0.05 vs. the C group; ^#^ *p* ≤ 0.05 vs. the CI group. Control, non-treated; CI, control injection; SI, H_2_S donor injection. Scale bar = 500 µm.

Histopathological alterations in the cartilage and synovial tissue at day 40 were analyzed by conventional staining methods. As expected, the cartilage from the medial compartment of the knee under surgical-induced OA showed pathological responses that were not detected in the medial compartment of sham-operated joints (Figure 3). Specifically, we detected a loss of safranin-o staining, an indicator of proteoglycan content, and the presence of vertical clefts/erosion, whose extension varied between the experimental groups (Figure 3a). When the analysis of the cartilage damage by the semi-quantitative modified osteoarthritis Research Society International (OARSI) score was performed, we observed that treatment with saline did not reduce the destruction of the cartilage in relation to that detected in the non-treated group (Figure 3b,c). However, the joints from the SI group showed a significantly lower damage in the cartilage from both the tibial plateau and femoral condyle in comparison with that observed in the control group. Additionally, the treatment with the H_2_S donor also attenuated destruction in the cartilage compared to that registered in the CI group, although these differences were only statistically significant in the tissue from femoral condyle (Figure 3b,c). Likewise, non-significant differences were detected in the cartilage from sham-operated joints between groups (Figure 3).

**Figure 4:**
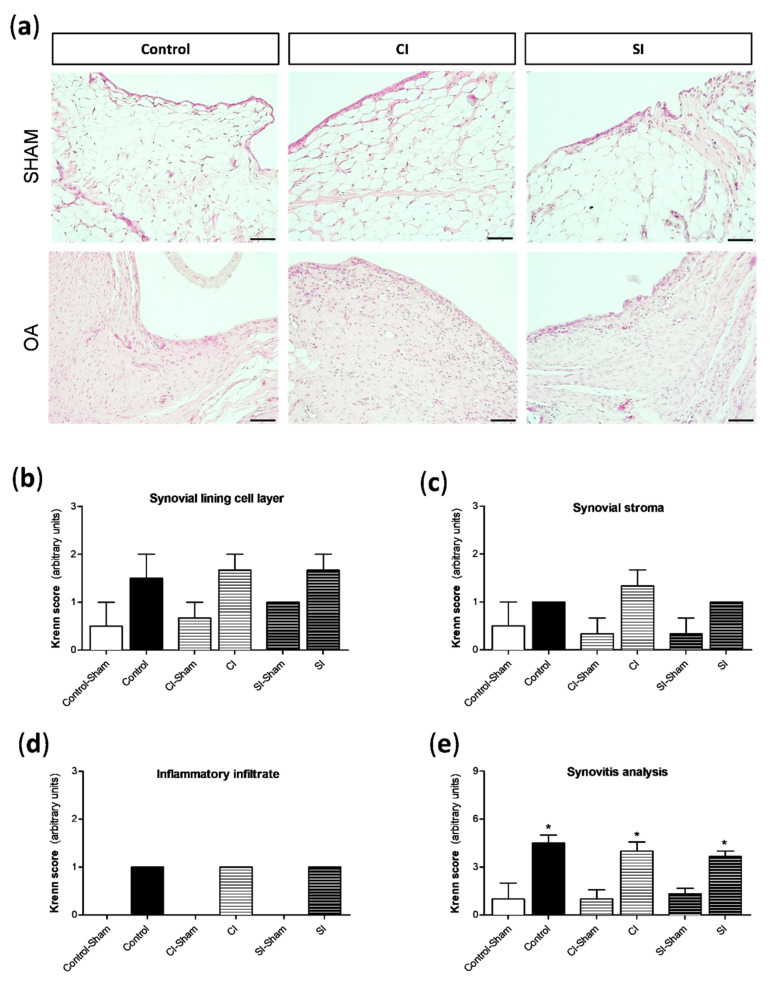
Histological analysis of synovial tissue. Pathological responses in the synovial tissue were evaluated by a semi-quantitative Krenn score, as previously indicated in the Materials and Methods section. (**a**) Representative images of synovial sections stained with hematoxylin and eosin from each group of the study, showing synovium in the medial compartment of the right knee (sham surgery) and left knee (OA surgery). Analysis of the semi-quantitative score of the following pathological alterations in the synovial tissue: number of lining cell layers (**b**), proliferation of the subintima tissue (**c**), and infiltration of inflammatory cells (**d**). (**e**) The sum up of the pathological changes in the tissue is shown. Values are mean ± SEM (*n* = 3 independent animals for each condition). * *p* ≤ 0.05 vs. the respective sham-operated joint. Control, non-treated; CI, control injection; SI, H_2_S donor injection. Scale bar = 50 µm.

The presence of pathological alterations in the synovial tissue was assessed by the semi-quantitative Krenn score (Figure 4). The surgical destabilization of the joint induced a clear increase in the number of lining cell layers (Figure 4b), a slight proliferation of subintima tissue (Figure 4c), and limited cell infiltration in the synovium from all the experimental groups (Figure 4d). Subsequently, we detected significant differences in the grade of synovitis between the operated and sham-operated joints (Figure 4e). However, no differences were found among the three groups.

### 2.3. Oxidative Damage Is Attenuated by Intraarticular Administration of H_2_S

**Figure 5:**
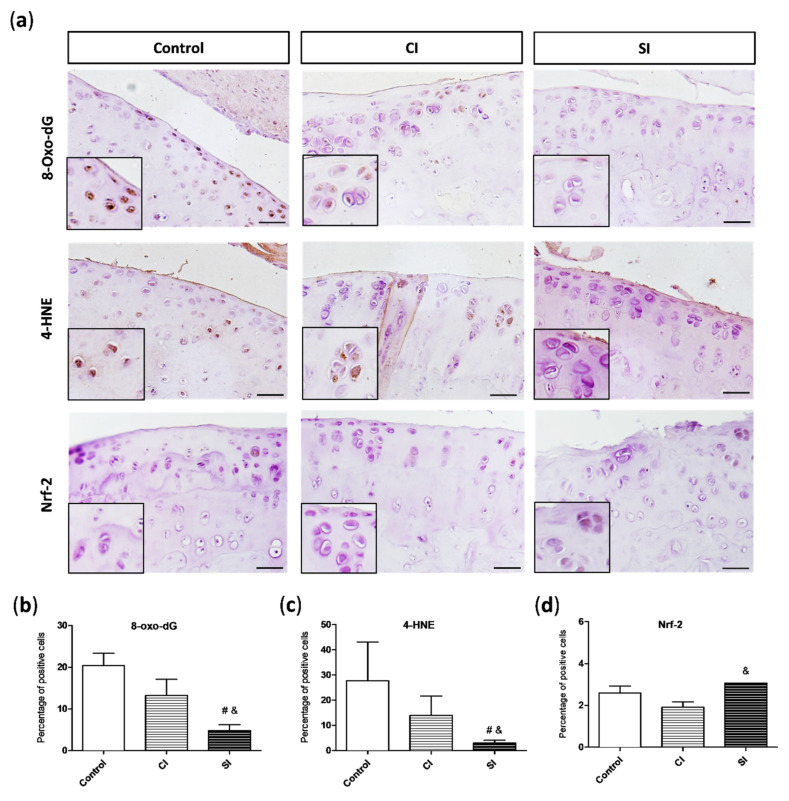
Presence of oxidative damage markers and antioxidant response in the articular cartilage. (**a**) Representative images of 8-hydroxy-2′-deoxyguanosine (8-oxo-dG), 4-hydroxy-2-nonenal (4-HNE), and nuclear factor erythroid-derived 2-like 2 (Nrf-2) immunohistochemistry in the cartilage from each group of the study. Magnification (2x) of the images are shown on its bottom-left corner. Quantitative analysis of 8-oxo-dG (**b**), 4-HNE (**c**), and Nrf-2 (**d**)-positive cells. Values are mean ± SEM (*n* = 3 independent animals for each condition). ^&^ *p* ≤ 0.05 vs. the C group; ^#^ *p* ≤ 0.05 vs. the CI group. Control, non-treated; CI, control injection; SI, H_2_S donor injection. Scale bar = 20 µm.

In the pathological scenario observed in the joint under the surgically induced OA, ROS overproduction exacerbates oxidative stress and may result in the induction of oxidative damage to DNA, proteins, and lipid membranes. Likewise, a growing number of studies support the pivotal role of oxidative damage in OA pathogenesis [3,4]. In order to evaluate the presence of oxidative damage in the cartilage, we assayed the levels of 8-hydroxy-2′-deoxyguanosine (8-oxo-dG), an indicator of DNA damage, and 4-hydroxy-2-nonenal (4-HNE), a maker of membrane lipid oxidation, by immunohistochemistry. As shown in Figure 5a, the experimental OA elicited an increment in the 8-oxo-dG levels in the joint. However, the cartilage from the SI group presented a significantly lower number of positive cells for this DNA oxidative maker than the cartilage from both control groups (Figure 5b). In a similar way, we also detected the presence of 4-HNE in the cartilage of all the experimental groups (Figure 5a), although the levels of this end product of lipid peroxidation were significantly higher in the control and CI group than in those treated with the slow-releasing H_2_S donor (Figure 5c).

One of the mechanisms of the chondrocytes to response to the increment in oxidative stress in the cartilage is the activation of Nrf-2 expression. We evaluated the levels of this transcription factor in the joint of the three experimental groups (Figure 5a). The presence of Nrf-2 in the cartilage was higher in the animals treated with H_2_S donor than in those from the control and CI groups, achieving significant differences in comparison with the Nrf-2 levels detected in the CI group (Figure 5d).

### 2.4. Intraarticular Injection of H_2_S Reduces Levels of Pro-catabolic Mediators in the Cartilage

**Figure 6:**
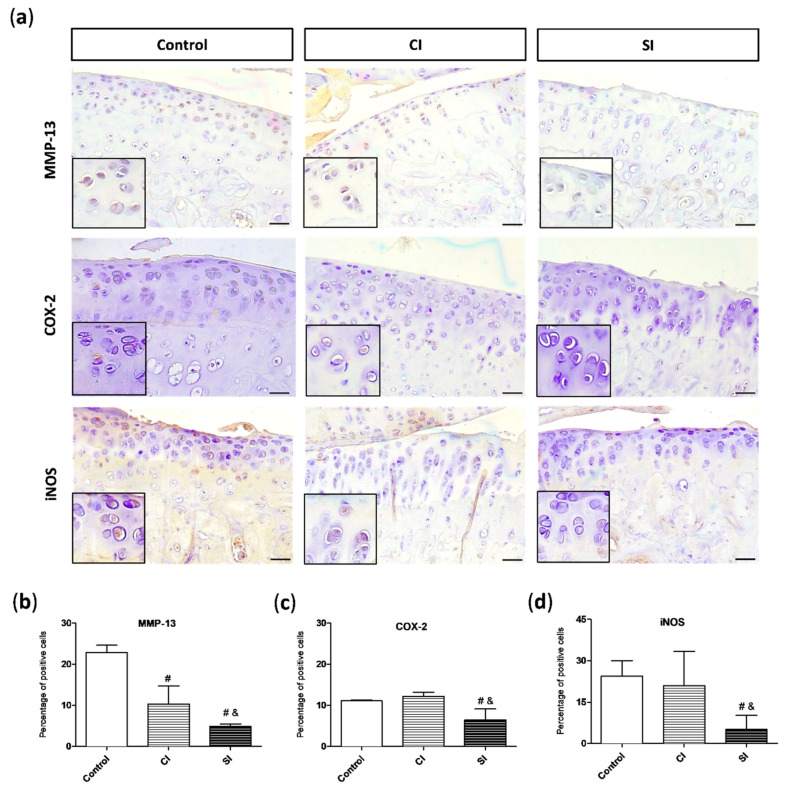
Presence of pro-catabolic mediators in the articular cartilage. (**a**) Representative images of metalloproteinase-13 (MMP-13), inducible nitric oxide synthase (iNOS), and cyclooxygenase 2 (COX-2) immunohistochemistry in the cartilage from each group of study. Magnifications (2x) of the images are shown on their bottom-left corners. Quantitative analysis of MMP-13 (**b**), iNOS (**c**), and COX-2 (**d**)-positive cells. Values are mean ± SEM (*n* = 3 independent animals for each condition). ^&^ *p* ≤ 0.05 vs. the C group; ^#^ *p* ≤ 0.05 vs. the CI group. Control, non-treated; CI, control injection; SI, H_2_S donor injection. Scale bar = 50 µm.

As previously mentioned, MMP-13 is one of the main enzymes involved in the degradation of cartilage ECM. When we analyzed the levels of this pro-catabolic mediator in the joint, we observed that the cartilage from the control group showed a significantly higher number of cells positive for MMP-13 than in the tissue from the CI and SI groups (Figure 6a,b). However, the levels of MMP-13 in the H_2_S injected group were significantly lower than those detected in the saline-injected animals (Figure 6b). The presence of COX-2 and iNOS, pro-inflammatory mediators commonly associated with the synthesis and release of MMP-13 among other pathways, were also evaluated (Figure 6c,d). As shown in the Figure 6, the levels of both markers in the cartilage from the SI group were significantly diminished in relation to those observed in the tissue from both the control and CI groups.

### 2.5. Blood Gene Expression of Mediators Involved in Antioxidant and Inflammatory Responses Is Modulated by the Intraarticular Administration of H_2_S

**Figure 7:**
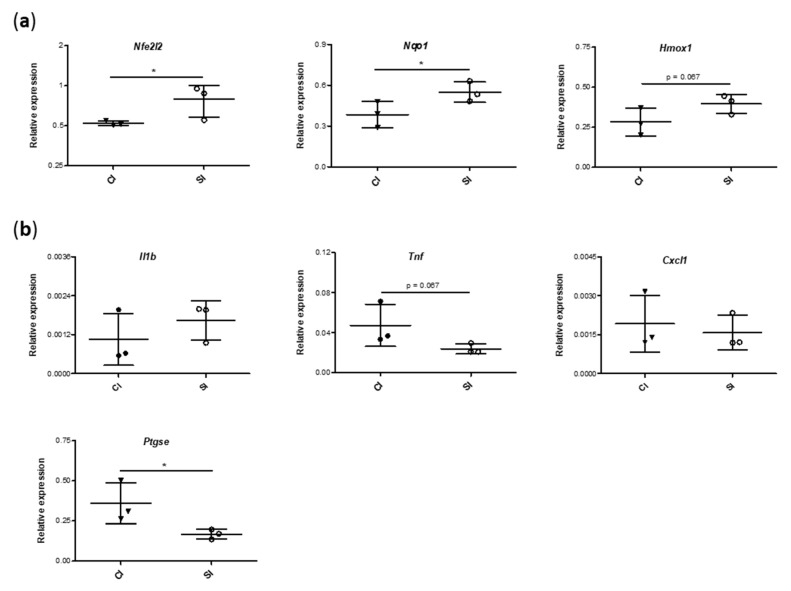
Measurement of the gene expression of antioxidant and pro-inflammatory markers. Relative expression levels of genes involved in antioxidant response (**a**) (nuclear factor erythroid-derived 2-like 2 (*Nfe2l2*), nicotinamide adenine dinucleotide phosphate hydrogen (NADPH):quinone oxidoreductase (*Nqo1*), and heme oxygenase-1 (*Hmox1*)) and in pro-inflammatory signaling (**b**) (interleukin-1β (*Il1b*), tumor necrosis factor-α (*Tnf*), cytokine-induced neutrophil chemoattractant-1 (*Cxcl1*), cyclooxygenase 2 (*Ptgse*)) were analyzed in blood samples from each group of the study, as previously indicated. Values are mean ± SEM (*n* = 3 independent animals for each condition). * *p* ≤ 0.05 vs. the CI group. CI, control injection; SI, H_2_S donor injection.

In order to further confirm our previous results and to elucidate the putative molecular mechanisms responsible for the protective effect of the exogenous administration of H_2_S, we analyzed the gene expression of Nrf-2, a master regulator of anti-oxidative responses, and NQO1 and HO-1, antioxidant enzymes whose expression in mainly modulated by this transcription factor. The expression of Nrf-2 (*Nfe2l2*), nicotinamide adenine dinucleotide phosphate hydrogen (NADPH):quinone oxidoreductase (*Nqo1*), and heme oxygenase-1 (*Hmox1*) genes in the blood from H_2_S-injected rats were higher than that from the control-injected animals, achieving statistically significant differences for the first two genes (Figure 7a). Then, we evaluated the expression of a number of pro-inflammatory genes commonly involved in pathological processes in the joint. As shown in the Figure 7b, the intraarticular administration of H_2_S reduced the levels of tumor necrosis factor-α (*Tnf*) and COX-2 (*Ptgse*) genes in relation to those observed in the CI group, with the modulation of ptgse expression being statistically significant. Conversely, we failed to observe any noteworthy differences in the levels of interleukin-1β (*Il1b*) and cytokine-induced neutrophil chemoattractant-1 (*Cxcl1*) between the experimental groups.

## 3. Discussion

OA is a multifactor chronic disease that causes pain and disability as, during its development, the entire joint organ is affected. There is no cure for OA, and treatment mainly involves drug intervention strategies in order to relieve pain and symptoms. Nonetheless, available structure-modifying agents show limited efficacy and, in some cases, adverse effects [31,32]. In the pursuit of an effective and alternative pharmacological treatment for this disease, H_2_S has emerged as a putative therapeutic candidate, showing a beneficial effect in OA [33]. However, the precise mechanisms of action of H_2_S have not been completely clarified yet. In this study, we described for the first time to our knowledge the effect of the intraarticular administration of GYY-4137, a slow-releasing H_2_S donor, in an in vivo experimental model of OA. Our results suggest a protective effect of the exogenous induction of H_2_S on joint pain and motor dysfunction, as well as cartilage destruction, likely as a result of the activation of antioxidant responses and the inhibition of catabolic signaling pathways in the joint.

A growing number of findings suggest that impaired H_2_S biosynthesis in the joint might be a contributing factor to OA [24,34]. We recently observed that OA cartilage shows reduced levels of the mitochondrial enzyme 3-Mercaptopyruvate sulfurtransferase (3-MPST) that could be responsible for the diminished H_2_S levels in this tissue [24]. Additionally, Nasi et al. (2020) described that the oxidative stress decreases the 3-MPST expression in the chondrocyte and in turn may reinforce the impairment of H_2_S biosynthesis in the OA joint [34]. Thus, in the current study the increment in oxidative stress after the surgical destabilization of the joint could be underlying a reduction in the H_2_S levels. Interestingly, the intraarticular administration of a H_2_S donor reduced oxidative damage, suggesting its protective impact on the maintenance of redox and H_2_S homeostasis in the joint. However, future experiments should elucidate whether GYY-4137 modulates H_2_S levels in the joint by inducing gas release [35,36], but also by modulating the expression of H_2_S-synthesizing enzymes, such as cystathionine γ-lyase (CSE), cystathionine β-synthetase (CBS), and MPST. In this sense, the effect of H_2_S donors on H_2_S-synthesizing enzymes is still unclear, as, for instance, different studies show contradictory results [37,38,39].

One of the main goals in OA management is focused on the reduction in pain and stiffness, and subsequently maintaining or improving physical functioning. In the current study, our results seem to indicate that the intraarticular administration of H_2_S reduces pain induced by the surgical destabilization of the joint, as well as recovering the joint flexibility and attenuating the impaired motor function detected by the Rotarod performance test to a higher extent than saline. This is despite the fact that the intraarticular injection of saline has also shown some beneficial effects, as it is now considered to be effective at alleviating nociceptive pain and in turn is not a true placebo in OA [40]. Accordingly, Batalle et al. (2019) observed that the systemic administration of slow-releasing H_2_S donors reduces mechanical allodynia and the grip strength deficits induced by the intraarticular injection of monosodium iodoacetate (MIA) in mice [27]. Lucarini et al. (2018) also demonstrated the antinociceptive effects of different compounds exhibiting slow H_2_S-release properties in an osteoarthritic pain model [41]. Similarly, we previously detected that balneotherapy with sulfurous-rich water was more effective against surgically induced OA, in terms of reduction in articular pain and improvement of physical response, than a bath in tap water [28].

The etiology of pain and disability in OA is complex and multifactorial. Pathological changes in the joint capsule and periarticular ligaments are a likely source [42,43]. Thus, one of the main mechanisms that characterizes this pathology and contributes to pain and stiffness is cartilage loss. We observed that intraarticular GYY-4137 administration protected cartilage against deterioration induced by the OA model. Previous studies support these findings [26,28,34]. For instance, the intra-articular injection of sodium hydrosulfide (NaSH), a fast-releasing H_2_S donor, slowed the development of degenerative changes in the articular cartilage in a model of gonarthrosis [26]. Likewise, cartilage damage as well as proteoglycan loss were exacerbated in 3-MST KO joints compared to WT joints in an experimental OA in mice [34]. By ex vivo and in vitro experimental approaches, a great number of studies also indicate the activation of H_2_S signaling as a protective pathway against cartilage break down. Hence, we have previously observed that H_2_S donors, including GYY-4137, reduced the activation of catabolic pathways in the cartilage, such as the expression of MMP-3 and-13 and the loss of cartilage extracellular matrix components [20]. Similarly, other studies have detected that NaSH inhibited the MMP-13 expression induced by inflammatory stimuli in chondrocytes [21,22]. Interestingly, we observed in the current study that H_2_S donor also attenuated the increment in MMP-13 levels in the cartilage elicited by the surgical destabilization of the joint. Overall, these findings further suggest the inhibition of this catabolic enzyme as an underlying mechanism responsible for the protective effect of H_2_S on cartilage destruction, although more studies will be necessary to elucidate this pathway.

Conversely, the intraarticular administration of H_2_S failed to show any modulation in the inflammation of the synovial tissue in our model. This is despite the fact that previous in vitro and in vivo studies have indicated that the induction of H_2_S biosynthesis attenuates the expression of pro-inflammatory mediators as well as pathological responses in the synovium [44,45,46]. However, most of these studies evaluated the early response to H_2_S administration, when an acute inflammatory reaction has taken place and, subsequently, the modulation of these responses is more easily detectable. Different studies have described that, in the model of the destabilization of the medial meniscus, an acute inflammatory response occurs very early after joint injury, which is sustained in the first two weeks and dropped at a lower level at the later phases, becoming a chronic low-grade inflammation [47,48] that mimics OA progression [1,3,49]. In agreement, we performed the histological analysis of synovial tissue at a later stage of the postramautic OA, so that all joints under surgical destabilization only showed a low grade of synovitis according to the grading system of Krenn [50], and likely differences between experimental conditions in terms of synovitis could be still indistinguishable between a phase of persistent chronic inflammation or in resolution. Future studies are warranted to further address this issue.

Different authors have observed in vitro that H_2_S presents antioxidant and anti-inflammatory effects on articular cells differently activated [21,22,44]. Here, we observed that the administration of H_2_S donor downregulated the levels of MMP-13, COX-2, and iNOS in the cartilage. Additionally, although the modulation of circulating pro-inflammatory makers in the joint was not evaluated in our study, we also detected a reduction in the gene expression of COX-2 and TNF-α at a systemic level under H_2_S treatment. In agreement, we have previously described that GYY-4137 decreased the expression of pro-inflammatory markers in chondrocytes by an in vitro experimental approach [19]. Besides this, other authors have also detected the capacity of H_2_S to reduce the levels of inflammatory mediators in the joint in in vivo studies [27] [44,46]. iNOS and COX-2 are enzymes responsible for the synthesis of NO and PGE_2_, respectively, which are widely known to be key mediators that contribute to the pain and pathogenesis of OA [6,51]. Tumor necrosis factor-α (TNF-α) is considered a pivotal cytokine in joint inflammation and hypernociception and initiates catabolic responses in the chondrocyte, including the generation of ROS and the activation of iNOS and COX-2 expression [10,52,53,54]. Interestingly, ATB-346, a novel H_2_S-releasing naproxen, more efficiently reduces TNF-α release in a rat model of arthritis [55], and GYY-4137 inhibits the expression of effectors of the TNF pathway [56]. Thus, the inhibition of the production of these mediators has been associated with the chondroprotective effect of H_2_S [27,55,56], and subsequently it could be responsible for the lesser OA severity and pain observed in our model under GYY-4137 treatment.

The effects of H_2_S are likely mediated through its capacity to inhibit nuclear factor-κB (NF-κB) signaling and promote Nrf-2 transcriptional activity [57]. Nrf-2 is a master regulator of antioxidant, anti-inflammatory, and other cytoprotective mechanisms. It regulates the expression of genes that code for phase II detoxification enzymes, such as NADPH:quinone oxidoreductase (NQO1) and heme oxygenase-1 (HO-1), which lead to amelioration of oxidative stress in cellular and animal models [58]. In our study, the intraarticular induction of H_2_S biosynthesis attenuated oxidative stress, reducing the presence in the cartilage of the markers of oxidative damage 4-HNE and 8-oxo-dG. Likewise, we observed that animals under H_2_S treatment showed an upregulated expression of Nrf-2, NQO1 and HO-1, suggesting that the activation of this signaling pathway could be involved in the antioxidant effect of H_2_S. These findings are in accordance with the fact that the downregulation of Nrf-2/HO-1 signaling increases OA severity, whereas its induction elicits protective actions in this pathology [59,60]. Nonetheless, we and other authors observed an upregulation of Nrf-2 levels in OA joint, likely as an insufficient response against the oxidative stress generated during this articular disorder [61,62]. Additionally, a recent study described that the expression of Nrf-2-regulated detoxicant enzymes, such as HO-1 and NQO1, is significantly increased in MIA-injected mice and that treatment with slow H_2_S-release donors maintains these high levels [27]. Wu et al. (2016) observed that S-propargyl-cysteine, an endogenous inductor of H_2_S synthesis, activates Nrf-2 signaling in adjuvant-induced arthritis rats and inhibits inflammatory response, ameliorating the severity of the arthritis model [46]. Hence, a growing number of evidences suggest that the Nrf-2 activation induced by H_2_S is also responsible for its anti-inflammatory effects [46,63,64]. In our study, we observed for the first time that the administration of a slow-releasing H_2_S donor in the joint induces the expression of Nrf-2 in the cartilage and that this event could contribute to the attenuation of the expression of pro-inflammatory markers detected in our model and subsequently to its pathogenesis. Nonetheless, future experiments should be encouraged to further enlighten us about the involvement of Nrf-2 in H_2_S actions in the joint.

## 4. Materials and Methods

### 4.1. Experimental Osteoarthritis in Rats

Eighteen female Wistar rats (Harlan Interfauna Ibérica, Barcelona, Spain) weighing between 350 and 450 g (8–10 months) were used. The animals were kept at room temperature (20–24 °C) and commercial food and water was available ad libitum. Experimental osteoarthritis was induced by the transection of the medial collateral ligament and the removal of the medial meniscus of the left joint, and the right joint was sham-operated and employed as a control [28]. Then, the animals were randomized into three groups (6 rats per group): Group 1 (Control, C), not treated; Group 2 (control injection, CI), intraarticularly injected with vehicle, saline; and Group 3 (Sulphide injection, SI), treated with a single intraarticular injection of the slow-releasing H_2_S donor GYY-4137 (morpholin-4-ium 4 methoxyphenyl(morpholino) phosphinodithioate) (Santa Cruz Biotechnology, Heidelberg, Germany) at 200 µM resuspended in 50 µL of saline [19,20,35,44]. Single injection was carried out at day 7 after surgery with a 29-gauge needle. Animals were euthanized at day 40. All the animal experiments were performed according to protocols approved by the Local Ethical Committee of Animal Experimentation (Comité de Ética de Experimentación Animal de la Xerencia de Xestión Integrada A Coruña (CEEA-XXIAC); 15002/2015/12) and the European Directive 2010/63, including treatment with antibiotics, and analgesics and painkiller drugs correspondingly.

### 4.2. Macroscopic and Clinical Evaluation

The macroscopic evaluation of the animals was performed at days 0 (before surgery), 7, 15, and 40. Firstly, the loss of weight was monitored and the joint width was measured by a digital caliper (S-CalWork, Sylvac, Malleray, Switzerland) in order to evaluate the joint swelling. The evaluation of pain levels was tested by the palpation of the knee and subsequently scoring the response of the rats to the joint manipulation on the 0 to 2 scale, with 0 being no response and 2 being the most painful response. Finally, the loss of joint flexion and extension angle were measured by a protractor.

### 4.3. Rotarod Performance Test

All the animals were subject to a Rotarod performed test (Ugo Basile S L, Varese, Italy) at days 0 (before surgery), 7, 15m and 40 after a training period of 2 weeks. Briefly, the rats were placed on a cylinder (rod) rotating at 30 rpm for 300 **s** and forced to move. Then, the number of falls during this period was registered, as well as the time that the rats stayed on the Rotarod before the first fall.

### 4.4. Histological Analysis

Knee joints from rats were the dissected, fixed in 4% formaldehyde in phosphate buffered saline (PBS), decalcified with DECALTM (Histolab, Askim, Norway), and embedded in paraffin (frontal section). Lesions in the synovial tissue and cartilage were evaluated by semi-quantitative analysis by two blinded researchers using an Olympus microscope (Olympus BX61, Olympus Biosystems, Barcelona, Spain). Sections (4-μm thick) of the joint were stained with hematoxylin and eosin, Masson’s trichrome, or Safranin O-fast green (Merck, Madrid, Spain). Two sections per animal were employed to evaluate damage score for cartilage and synovial tissues. According to the semi-quantitative modified OARSI score, the grade of the cartilage lesion was scored from 0 to 5 (with 0 being not damaged and 5 being most damaged) [65]. The grade of the synovial lesion was scored from 0 to 9 (0 being not damaged and 9 most damaged), and the evaluation parameters were the numbers of lining cell layers, the proliferation of the subintima tissue, and the infiltration of inflammatory cells [50].

### 4.5. Immunohistochemistry

The MMP-13, 8-oxo-dG, 4-HNE, Nrf-2, COX-2, and iNOS levels were evaluated on sections from the paraffin-embedded joints of rats. Slides were first deparaffinized with xylene and then rehydrated in graded ethanol and water. Heat-mediated antigen retrieval was performed in citrate buffer (pH 6.0; Dako, Glostrup, Denmark) for MMP-13, Nrf-2, and iNOS detection or in ethylenediaminetetra-acetic acid (EDTA) buffer (pH 9.0; Dako) for 4-HNE detection. Endogenous peroxidase activity was quenched using a commercial reactive (Dako) for 10 min. After washing in PBS, the slides were incubated 1 h at room temperature with anti-8-oxo-dG (1:200, Abcam, Cambridge, UK), anti-4-HNE (1:150, Abcam), and anti-COX-2 (1:200, Santa Cruz Biotechnology) antibodies, or anti-MMP-13 antibody (1:100, Abcam) after blocking with 20% normal goat serum for 10 min. Nrf-2 (1:200, Santa Cruz Biotechnology) and iNOS (1:200, Abcam) antibodies were incubated overnight. Bound antibodies were detected with a goat secondary antibody (ready-to-use; Dako) and diaminobenzidine using the commercial EnVision™ Detection System (Dako). Finally, sections were counterstained with Gill III hematoxylin (Merck) and mounted with DePeX (Sigma). Three images of each slide (two slides per animal) were captured with a computer-controlled digital camera (Olympus BX61, Olympus). Using image processing software (Image J software, http://imagej.nih.gov/), the percentage of chondrocyes presenting a positive signal of immunostaining in the cartilage was calculated and represented as the ratio of positive cells from the total cells observed in the area of cartilage captured in the images.

### 4.6. RNA Analysis

Blood samples were collected at day 40 from 3 animals per experimental group. Subsequently, RNA was extracted with the commercial kit Mouse RiboPureTM (Thermo Fisher Scientific, Vilnius, Lithuania) following the manufacturer’s instructions. Then, cDNA was generated by the reverse-transcription of total RNA (500 ng) with the NZY First-Strand cDNA Synthesis kit (NZYTech, Lisbon, Portugal). The levels of interleukin-1β, TNF-α, cytokine-induced neutrophil chemoattractant-1, COX-2, Nrf-2, HO-1, and NQO1 mRNAs were quantified using a Light Cycler LC480 (Roche Diagnostics). The relative mRNA expression levels were calculated and normalized to the levels of the rat hypoxanthine guanine phosphoribosyltransferase mRNA using the 2 CT method (specific rat primer sequences are shown in Table 1). All the measurements were performed in duplicate.

### 4.7. Statistical Analysis

Data are presented as the mean ± standard error of the mean (SEM) or as representative results, as indicated. The GraphPad PRISM version 5 statistical software package (La Jolla, CA, USA) was used to perform repeated ANOVA, followed by Bonferroni’s post-hoc comparisons test. Additionally, statistically significant differences between the experimental groups were determined by an unpaired comparison test. *p* ≤ 0.05 was considered statistically significant.

## 5. Conclusions

Our results indicate a beneficial effect of the intraarticular induction of H_2_S biosynthesis on experimental OA. The administration of a H_2_S donor in the joint promoted an attenuation in the pain and severity of articular damage and inflammation, as well as a reduction in oxidative damage after the surgical destabilization of the joint. These effects seem to be mediated, at least partially, through the activation of Nrf-2 and subsequently the expression of antioxidant enzymes, such as HO-1 and NQO1. Accordingly, new non-steroidal anti-inflammatory drugs with H_2_S-releasing additional properties are being developed. For instance, ATB-346 has shown a higher capacity to attenuate OA pathogenesis in experimental assays [55,66], and it has recently been demonstrated to be a more efficient and safer alternative to existing nonsteroidal anti-inflammatory drugs (NSAIDs) in a Phase 2 clinical trial [67]. Nowadays, it is widely accepted that the future of OA treatment may lie in combination therapy. Thus, the findings presented in the current study further reinforce the use of alternative molecules showing H_2_S-release properties as a complementary intervention in the management of rheumatic diseases such as OA.

## Figures and Tables

**Table 1:** Sequences of the primers used to analyze gene expression.

Name	Forward	Reverse
Interleukin-1β (*Il1b*)	gctgacagaccccaaaagat	agctggatgctctcatctgg
Tumor necrosis factor-α (*Tnf*)	gcccagaccctcacactc	ccactccagctgctcctct
Cytokine-induced neutrophil chemoattractant-1 (*Cxcl1*)	cacactccaacagagcacca	tgacagcgcagctcattg
Cyclooxygenase 2 (*Ptgse*)	atgacgagcgactgttcca	tcaggtgttgcacgtagtcttc
Nuclear factor erythroid-derived 2-like 2 (*Nfe2l2*)	acgtgatgaggatgggaaac	gctcttgggaacaaggaaca
Heme oxygenase-1 *(Hmox1*)	gaaagcttttggggttcctc	gcctctaccgaccacagttc
NADPH:quinone oxidoreductase (*Nqo1*)	ctttctgtgggccatcattt	gaggcccctaatctgacctc
Hypoxanthine guanine phosphoribosyltransferase 1 (*Hprt1*)	gaccggttctgtcatgtcg	acctggttcatcatcactaatac

## Abbreviations

OA	osteoarthritis
H_2_S	hydrogen sulfide
Nrf-2	nuclear factor-erythroid 2-related factor 2
PGs	prostaglandins
MMPs	metalloproteinases
COX-2	inducible isoform of cyclooxygenase
ROS	reactive oxygen species
NO	nitric oxide
iNOS	inducible NO synthase
ECM	extracellular matrix
C	group under non-treatment
CI	group treated with intraarticular injection of saline
SI	group treated with intraarticular injection of H_2_S donor
MTP	medial compartment from tibial plateau
MFC	medial compartment from femoral condyl
8-oxo-dG	8-Hydroxy-2′-deoxyguanosine
4-HNE	4-hidroxi-2-nonenal
*Nfe2l2*	nrf-2 gene
*Nqo1*	NADPH:quinone oxidoreductase gene
*Hmox1*	heme oxygenase-1 gene
*Tnf*	tumor necrosis factor-α gene
*Ptgse*	COX-2 gene
*Il1b*	interleukin-1β gene
*Cxcl1*	cytokine-induced neutrophil chemoattractant-1 gene
3-MPST	3-Mercaptopyruvate sulfurtransferase
CSE	cystathionine γ-lyase
CBS	cystathionine β-synthetase
MIA	monosodium iodoacetate
NaSH	sodium hydrosulfide
TNF-α	tumor necrosis factor-α
NF-κB	nuclear factor-κB
NQO1	NADPH:quinone oxidoreductase
HO-1	heme oxygenase-1
SEM	standard error of the mean
